# Health inequalities by socioeconomic characteristics in Spain: the economic crisis effect

**DOI:** 10.1186/s12939-016-0346-4

**Published:** 2016-04-11

**Authors:** Clara Barroso, Ignacio Abásolo, José J. Cáceres

**Affiliations:** Departamento de Economía Aplicada y Métodos Cuantitativos, Facultad de Economía, Empresa y Turismo, Universidad de La Laguna, Campus de Guajara, 38071 La Laguna, Tenerife Spain

**Keywords:** Health status, Socioeconomic characteristics, Health inequalities, Economic recession, Spain

## Abstract

**Background:**

An economic crisis can widen health inequalities between individuals. The aim of this paper is to explore differences in the effect of socioeconomic characteristics on Spaniards’ self-assessed health status, depending on the Spanish economic situation.

**Methods:**

Data from the 2006–2007 and 2011–2012 National Health Surveys were used and binary logit and probit models were estimated to approximate the effects of socioeconomic characteristics on the likelihood to report good health.

**Results:**

The difference between high and low education levels leads to differences in the likelihood to report good health of 16.00–16.25 and 18.15–18.22 percentage points in 2006–07 and 2011–12, respectively. In these two periods, the difference between employees and unemployed is 5.24–5.40 and 4.60–4.90 percentage points, respectively. Additionally, the difference between people who live in households with better socioeconomic conditions and those who are in worse situation reaches 5.37–5.46 and 3.63–3.74 percentage points for the same periods, respectively.

**Conclusions:**

The magnitude of the contribution of socioeconomic characteristics to health inequalities changes with the economic cycle; but this effect is different depending on the socioeconomic characteristics indicator that is being measured. In recessive periods, health inequalities due to education level increase, but those linked to individual professional status and household living conditions are attenuated. When the joint effects of individuals’ characteristics are considered, the economic crisis brings about a slight increase in the inequalities in the probability of reporting good health between the two extreme profiles of individuals. The design of public policies aimed at preventing any worsening of health inequalities during recession periods should take into account these differential effects of socioeconomic characteristics indicators on health inequalities.

## Background

The World Health Organization has affirmed the existence of a social gradient in health that corresponds to socioeconomic level [1], which has also been observed in many studies [2–7]. Insofar as an individual’s socioeconomic level is reduced, her health status is worsened. Therefore, factors as wealth, education, occupation or social conditions of place of residence, have an effect on health and illness; as a consequence, those more advantaged social groups experience greater health improvements [8, 9]. People are not indifferent regarding socioeconomic inequalities in health. Some studies have analysed this by exploring how members of the public perceive the balance of the objective of reducing health inequalities and the objective of improving average population health [10–12]; all of them show that there is a social concern for health inequalities in addition to health maximization. In Spain, a related study shows that the majority of the general population (around 69 %) supports policies that reduce socioeconomic inequalities in health (being younger and older individuals less likely to support egalitarian policies than those in the middle age) [13]. In addition, when eliciting public preferences concerning trade-offs between the total level of health and its distribution by socioeconomic groups, evidence from Spain shows that the majority of individuals give priority to health programmes that reduce socioeconomic health inequalities even when this implies an overall health loss to the population [14].

Health inequalities have always been present in the political agenda of the EU member states [15] and Spain has not been an exception. In 2008, the Spanish National Commission on the reduction of social health inequalities was created with the aim of proposing future public policies and other interventions to reduce social health inequalities in Spain. In addition, Spanish health regulations also include these targets: The General Health Act (1986) and the National Health System Cohesion and Quality Act (2003) aim to overcome health inequalities and guarantee equality of access to public health care services. The General Public Health Act (2011) also takes into account the social determinants of health. However, despite the aim of reduction of health inequalities is nowadays incorporated in governments’ agenda of most developed countries, they seem to remain generation after generation [8, 15]. Determinants of health inequalities are subjects to variations with social, economic, political and demographic changes. The most important change that has taken place at a global scale in the last years has been the economic and financial crisis. Some countries have recovered relatively soon but other countries, like Spain, are still suffering it.

Thus, the research question of this paper is whether the assumed relationship between socioeconomic level and health changes in recessive periods and, therefore, whether the economic crisis has widened existing socioeconomic health inequalities. Health is approached through Self Assessed Health (SAH), a measure widely used in the related literature. While the relationship between unemployment and health loss is well established in the related literature [16–20], some authors have found that the effect of unemployment on SAH does not significantly differ in recessive periods, with respect to expansive periods [20]. Other authors, however, find that this deterioration is intensified during times of crisis [16, 19]; although, according to the study of Astell and Feng, employees’ health also deteriorates in recessive periods [19]. The Spanish Society for Public Health and Health Administration (SESPAS) states that the negative impact of unemployment on health is emphasized among those without access to health care benefits, but employees’ health also deteriorates due to job insecurity, poorer working conditions, long-lasting stress, etc. [21].

The main objective of this paper is therefore to explore differences in the effect of several socioeconomic characteristics on Spaniards’ self-assessed health in both expansive and recessive periods of the economy. The empirical basis and the methodology are presented in the second section. The results are shown in the third section. And the fourth section presents the discussion and conclusions.

## Methods

The data used come from the 2006–2007 and the 2011–12 National Health Surveys (NHS), conducted by the National Statistics Institute (INE) jointly with the Spanish Ministry of Health, Social Services and Equality (MSSSI), and which employ a multistage, stratified-random design to identify the samples of adults of both Surveys (more details of the methodology of the Surveys can be found elsewhere [22, 23]. A total of 29,478 and 20,884 adult individuals have taken part in these surveys, respectively. After eliminating missing observations in both surveys and dropping the 15-year-old individuals from the 2011–2012 NHS (the 2006–07 survey for adults only included individuals aged 16 or over), the usable sample size is 29,272 and 20,841 individuals aged 16 or over in 2006–07 and 2011–12, respectively. Results have been obtained applying the corresponding weighting factors assigned to the individuals of the microdata.

The variable that represents the individual health status is Self-Assessed Health (SAH), a health measure widely used in sociological, epidemiological, medical and economic studies [18–20, 24–26]. Specifically, both National Health Surveys included the following question*: in the last twelve months, would you say your health status has been very good, good, fair, bad or very bad?* In line with other studies [18, 20, 24, 27], we have grouped the alternatives “very good” and “good” in the category *good*, while “fair”, “bad” and “very bad” alternatives have been grouped in the category *not good*. Thus, the problem caused by the heterogeneity of different personal views on SAH is attenuated. A dichotomous variable SAH_i_ has been defined for each individual, taking a value of 1 if the self-assessed health status by the *i-th* individual is good and 0 otherwise.

Regarding the vector of covariates and, in particular, the socioeconomic characteristics –the main focus of this paper-, we first have considered the interviewee’s economic situation. Household income was ruled out for two reasons: first, regarding income values, reference intervals are very different between both surveys (2006–07 and 2011–12) so comparison of both surveys can lead to biased results and, second, income is a variable with a very high percentage of missing observations. So, instead, the interviewee’s economic situation has been approximated through two attributes: an attribute indicating interviewee’s professional status (employee, unemployed, retired, student and others) and the other attribute tries to reflect household’s economic situation of the interviewee. To do this, using the household questionnaire, we have selected the information related to the reference person’s professional status, i.e. the largest contributor to the household budget. Additionally, it has been assumed that households where the reference person works are in a better economic situation than those where the reference person does not work. A third socioeconomic characteristic taken into account has been the interviewee’s educational level, for which we have considered three categories: no education or primary school, secondary studies and university studies.

Another two covariates that have been considered crucial in the analysis are age and gender. First, age is an important predictor of health and there are noticeable differences in average age between population groups defined by different socioeconomic and sociodemographic profiles; age is categorized by means of five dummies depending on the interviewee’s age group (16–34, 35–49, 50–64, 65–74 and 75 or more years). Second, men and women do not play the same role in society; in most industrialized countries, women are discriminated by the labour markets, having to perform less qualified jobs or devoting their time to look after other members in need of care within households [28]. Thus, several studies conclude that women have worse health status than men [18, 29, 30]. There is also evidence that each gender behaves in different ways regarding risk attitudes that may affect health; for instance, tobacco and alcohol consumption, risky behaviour regarding driving, or delay visits to specialist doctors when needed, are more frequent among men [28]. The dummy variable gender takes the value one for men and zero for women.

Other control explanatory variables included are marital status, that enters by means of a categorical variable (single, married or widowed), and a dichotomous dummy variable representing whether the individual is Spanish (or foreign). Table 1 shows the variables considered in the analysis, the description of the alternatives of each attribute and their main statistics, both for the 2006–07 and for the 2011–12 samples (the table also shows the average age for each group).

**Table 1:** Sample description

		2006–07		2011–12	
Attribute	Alternative	%^(1)^	Age^(2)^	%^(1)^	Age^(2)^
Self-assessed health	Good	66.50 (66.48, 66.51)	41.29	71.77 (71.75, 71.78)	42.94
	Not good	33.50 (33.49, 33.52)	55.37	28.23 (28.22, 28.25)	58.53
Education level	No education or primary school	41.93 (41.91, 41.94)	56.55	22.56 (22.54, 22.57)	62.75
	Secondary studies	41.10 (41.08, 41.11)	37.48	61.49 (61.48, 61.51)	42.91
	University studies	16.98 (16.97, 16.99)	40.64	15.95 (15.94, 15.96)	42.62
Interviewee’s professional status	Employee	51.48 (51.46, 51.49)	38.89	45.01 (44.99, 45.02)	41.40
	Unemployed	7.17 (7.16, 7.18)	37.19	14.60 (14.59, 14.61)	38.57
	Retired	20.60 (20.59, 20.61)	70.65	20.27 (20.26, 20.28)	73.31
	Student	6.58 (6.57, 6.59)	19.95	7.70 (7.69, 7.71)	20.17
	Others	14.17 (14.16, 14.18)	52.62	12.43 (12.42, 12.44)	53.60
Reference person professional status	Employee	67.13 (67.11, 67.14)	39.17	58.28 (58.27, 58.30)	40.61
	No employee	32.87 (32.86, 32.89)	59.98	41.72 (41.70, 41.73)	56.74
Gender	Man	49.08 (49.07, 49.10)	44.85	48.76 (48.75, 48.78)	46.18
	Woman	50.92 (50.90, 50.93)	47.13	51.24 (51.22, 51.25)	48.45
Age	16–34 years old	32.83 (32.81, 32.84)	26.05	28.68 (28.67, 28.70)	26.13
	35–49 years old	28.08 (28.06, 28.09)	42.04	29.25 (29.23, 29.26)	41.84
	50–64 years old	19.66 (19.65, 19.67)	56.68	21.61 (21.60, 21.62)	56.50
	65–74 years old	10.31 (10.30, 10.32)	69.47	10.28 (10.27, 10.29)	69.14
	75 or more years old	9.12 (9.11, 9.13)	80.55	10.18 (10.17, 10.19)	81.42
Nationality	Spanish	89.31 (89.30, 89.32)	47.26	88.04 (88.03, 88.05)	48.55
	Foreign	10.69 (10.68, 10.70)	35.56	11.96 (11.95, 11.97)	38.41
Marital status	Single and not living with a partner	25.59 (25.57, 25.60)	30.25	26.14 (26.13, 26.15)	31.58
	Married or single and living with a partner	63.24 (63.22, 63.25)	49.22	61.65 (61.63, 61.66)	50.54
	Widower, legally separated or divorced	11.17 (11.16, 11.18)	63.95	12.21 (12.20, 12.22)	64.91

^(1)^The extremes of corresponding 95 % confidence intervals are indicated in parentheses

^(2)^Average age in each group

Since the aim of this study is to approximate the effects of individual characteristics on interviewees’ SAH, we have taken into account the qualitative nature of the variable that we want to explain and have used a discrete choice model that allows the conjoint probabilistic quantification of such effects. Specifically, a binary logit model has been estimated for 2006–07 and another one for 2011–12. That is, we have estimated models as the following:

$$ P\left(SA{H}_i=1\right)=\frac{e^{\beta^{\mathit{\hbox{'}}}{x}_i}}{1+{e}^{\beta^{\mathit{\hbox{'}}}{x}_i}}, $$

where *SAH*_*i*_ = 1 if the self-assessed health status by individual *i* is good, *x*_*i*_ is the explanatory variables’ vector for individual *i*, and *β* is the parameters’ vector that determines the influence of these variables on the probability of reporting good health status. Furthermore, probit models,

$$ P\left(SA{H}_i=1\right)={\displaystyle {\int}_{-\infty}^{\beta^{\mathit{\hbox{'}}}{x}_i}\frac{1}{\sqrt{2\pi }}{e}^{-\frac{1}{2}{z}^2}dz}, $$

have been also estimated. The explanatory variables included in the chosen specification are dummy variables corresponding to socioeconomic and socio-demographic attributes. The formulation of these models facilitates the interpretation of the effects that we want to evaluate through simple calculations, such as *discrete changes*. These are changes in the predicted average probabilities when individual characteristics change, and *odds-ratios*, denoted by *Ω*_*i*/*j*_ and defined as the quotient between the ratio of predicted probabilities of reporting good health and bad health when the individual characteristics vector is *x*_*i*_, and the same ratio if the characteristics vector is *x*_*j*_ (see Appendix) [31]. This statistical analysis has been performed using Stata version 13 and SPSS (Statistical Package for the Social Sciences) version 20.0.

## Results

The relative frequencies included in Table 1 show important changes in the relative weights of the categories of socioeconomic attributes. The economic recession has reduced the proportion of respondents who work by 6.48 percentage points, and it has increased the percentage of unemployed individuals, which has doubled. Similarly, the percentage of respondents who live in households where the reference person works has fallen by 8.85 percentage points. The percentage of individuals who do not have education or have only primary school level has also fallen by slightly less than a half, while the percentage of individuals who have secondary school studies has multiplied by 1.5. However, the proportion of individuals who report good health in 2011–12 is 5.27 percentage points higher than in 2006–07.

Through the frequency two-dimensional analysis shown in Table 2, some interactions related to the effect of individuals’ socioeconomic characteristics on the propensity to report good health can be observed. The difference in health between individuals with university studies and individuals with no education or primary school is about 30.80 and 38.55 percentage points in 2006–07 and 2011–12, respectively. The percentage of those interviewees who work and report good health exceeds the corresponding percentage of those unemployed by 9.44 and 6.71 percentage points in both periods considered, respectively. Furthermore, the proportion of individuals who live in households where the reference person works and report good health is 25.08 and 22.40 percentage points higher than those who live with an unemployed reference person in each period. Regarding gender, the percentage of men who report good health exceeds the corresponding percentage of women by 11.97 and 9.21 percentage points in 2006–07 and 2011–12, respectively.

**Table 2:** Proportion of individuals who report good health

Attribute	Alternative	2006–07 (%)^(1)^	2011–12 (%)^(1)^
Education level	No education or primary school	51.24 (51.22, 51.26)	48.19 (48.17, 48.20)
	Secondary studies	75.64 (75.62, 75.65)	76.53 (76.52, 76.55)
	University studies	82.04 (82.03, 82.05)	86.74 (86.73, 86.75)
Interviewee’s professional status	Employee	77.05 (77.04, 77.06)	82.91 (82.90, 82.93)
	Unemployed	67.61 (67.60, 67.63)	76.20 (76.19, 76.21)
	Retired	40.71 (40.69, 40.72)	46.53 (46.51, 46.54)
	Student	91.50 (91.49, 91.51)	93.32 (93.31, 93.33)
	Others	53.47 (53.45, 53.48)	54.01 (54.00, 54.03)
Reference person professional status	Employee	74.74 (74.72, 74.75)	81.11 (81.10, 81.12)
	No employee	49.66 (49.64, 49.68)	58.71 (58.70, 58.73)
Gender	Man	72.59 (72.58, 72.60)	76.48 (76.47, 76.50)
	Woman	60.62 (60.60, 60.64)	67.28 (67.26, 67.29)
Age	16–34 years old	83.54 (83.53, 83.55)	89.40 (89.39, 89.41)
	35–49 years old	72.86 (72.85, 72.88)	78.60 (78.58, 78.61)
	50–64 years old	55.48 (55.46, 55.50)	65.28 (65.26, 65.29)
	65–74 years old	44.31 (44.30, 44.33)	53.94 (53.93, 53.96)
	75 or more years old	34.38 (34.36, 34.39)	34.23 (34.22, 34.25)
Nationality	Spanish	65.99 (65.98, 66.01)	70.96 (70.94, 70.97)
	Foreign	70.71 (70.70, 70.73)	77.73 (77.71, 77.74)
Marital status	Single and not living with a partner	81.15 (81.14, 81.16)	84.11 (84.10, 84.12)
	Married or single and living with a partner	64.20 (64.18, 64.21)	70.73 (70.72, 70.75)
	Widower, legally separated or divorced	45.94 (45.92, 45.96)	50.57 (50.56, 50.59)
Total		66.50 (66.48, 66.51)	71.77 (71.75, 71.78)

^(1)^The extremes of corresponding 95 % confidence intervals are indicated in parentheses

Nevertheless, the estimation of logit or probit models is more appropriate to quantify the above effects conjointly. However, a note of caution is needed about such interactions because they may be affected by indirect effects. In such a sense, as observed in Table 1, there are noticeable differences in average age between population groups defined by different socioeconomic and sociodemographic profiles. A first result obtained from the estimation of both models indicates the existence of a significant increase in the likelihood of reporting good health in 2011–12 compared to 2006–07 (as also observed in Tables 1 and 2). Tables 3 and 4 shows the estimated results of the models for the two periods considered. From these estimates we have obtained predicted average probabilities, and we have calculated discrete changes, which are included in Table 5 and odds-ratios that are included in Table 6.

**Table 3:** Estimates for binary logit models

Attribute	Category ^(1)^	2006–07^(2, 3)^	2011–12^(2, 3)^
Independent term		0.3695 (0.3645, 0.3745)	0.5852 (0.5799, 0.5905)
Education level	No education or primary school	−0.8728 (−0.8752, −0.8703)	−1.0891 (−1.0920, −1.0861)
	Secondary studies	−0.4617 (−0.4641, −0.4592)	−0.5973 (−0.5999,-0.5947)
Interviewee’s professional status	Unemployed	−0.2748 (−0.2779, −0.2718)	−0.2884 (−0.2911, −0.2857)
	Retired	−0.6081 (−0.6112, −0.6051)	−0.4855 (−0.4890, −0.4820)
	Student	0.7227 (0.7177, 0.7277)	0.5460 (0.5407, 0.5513)
	Others	−0.2318 (−0.2343, −0.2292)	−0.6766 (−0.6793, −0.6738)
Reference person professional status	No employee	−0.2793 (−0.2814, −0.2771)	−0.2153 (−0.2175, −0.2132)
Gender	Man	0.4863 (0.4846,0.4880)	0.2966 (0.2949, 0.2983)
Age	16–34 years old	1.0926 (1.0885, 1.0966)	1.7578 (1.7534, 1.7622)
	35–49 years old	0.6631 (0.6594, 0.6668)	1.0356 (1.0316, 1.0395)
	50–64 years old	0.1954 (0.1921, 0.1987)	0.6201 (0.6167, 0.6236)
	65–74 years old	0.3337 (0.3306, 0.3368)	0.6514 (0.6484, 0.6543)
Nationality	Spanish	0.3047 (0.3022, 0.3072)	0.1248 (0.1222, 0.1273)
Marital status	Single and not living with a partner	0.2122 (0.2090, 0.2154)	0.1700 (0.1669, 0.1732)
	Married or single and living with a partner	0.0771 (0.0746, 0.0795)	0.2132 (0.2108, 0.2155)
Goodness of fit measures^(4)^	Log likelihood ratio (15)	6332611.61	7109743.62
	McFadden’s R2	0.134	0.155
	Cragg & Uhler’s (Nagelkerke) R2	1	1
	McKelvey and Zavoina’s R2	0.997	0.998
	AIC	1392.738	1853.515
	BIC	4.05E + 07	3.84E + 07

^(1)^The reference category for each attribute is not shown

^(2)^The extremes of corresponding 95 % confidence intervals are indicated in parentheses

^(3)^All parameters are statistically significant at the 0.01 level

^(4)^All of these measures are obtained from Stata 13 (see Long and Freese, 2008:109–113)

**Table 4:** Estimates for binary probit models

Attribute	Category^(1)^	2006–07^(2, 3)^	2011–12^(2, 3)^
Independent term		0.2049 (0.2019, 0.2079)	0.3117 (0.3085, 0.3148)
Education level	No education or primary school	−0.5096 (−0.5111, −0.5082)	−0.6310 (−0.6327, −0.6293)
	Secondary studies	−0.2605 (−0.2619, −0.2591)	−0.3294 (−0.3309, −0.3280)
Interviewee’s professional status	Unemployed	−0.1596 (−0.1614, −0.1578)	−0.1586 (−0.1602, −0.1571)
	Retired	−0.3716 (−0.3735, −0.3697)	−0.2863 (−0.2884, −0.2842)
	Student	0.3859 (0.3833, 0.3885)	0.2733 (0.2707, 0.2759)
	Others	−0.1463 (−0.1479, −0.1448)	−0.4020 (−0.4036, −0.4003)
Reference person professional status	No employee	−0.1696 (−0.1709, −0.1683)	−0.1298 (−0.1310, −0.1285)
Gender	Man	0.2892 (0.2882, 0.2902)	0.1786 (0.1776, 0.1795)
Age	16–34 years old	0.6568 (0.6544, 0.6593)	1.0301 (1.0275, 1.0327)
	35–49 years old	0.4109 (0.4086, 0.4131)	0.6348 (0.6325, 0.6372)
	50–64 years old	0.1264 (0.1244, 0.1284)	0.3887 (0.3866, 0.3909)
	65–74 years old	0.2056 (0.2037, 0.2075)	0.4023 (0.4004, 0.4041)
Nationality	Spanish	0.1800 (0.1785, 0.1814)	0.0728 (0.0714, 0.0743)
Marital status	Single and not living with a partner	0.1301 (0.1282, 0.1320)	0.1167 (0.1148, 0.1185)
	Married or single and living with a partner	0.0529 (0.0515, 0.0544)	0.1326 (0.1311, 0.1340)
Goodness of fit measures^(4)^	Log likelihood ratio (15)	6330520.28	7112977.38
	McFadden’s R2	0.134	0.156
	Cragg & Uhler’s (Nagelkerke) R2	1	1
	McKelvey and Zavoina’s R2	0.998	0.999
	AIC	1392.81	1853.36
	BIC	4.05E + 07	3.84E + 07

^(1)^The reference category for each attribute is not shown

^(2)^The extremes of corresponding 95 % confidence intervals are indicated in parentheses

^(3)^All parameters are statistically significant at the 0.01 level

^(4)^All of these measures are obtained from Stata 13 (see Long and Freese, 2008:109–113)

**Table 5:** Average predicted probabilities to report good health from logit and probit models

		2006–07 (%)^(1)^		2011–12 (%)^(1)^	
Attribute	Alternative	Logit	Probit	Logit	Probit
Education level	No education or primary school	60.88	60.78	63.83	63.53
	Secondary studies	69.07	69.05	72.95	72.99
	University studies	77.12	76.77	82.05	81.69
Interviewee’s professional status	Employee	70.10	70.06	76.18	76.03
	Unemployed	64.70	64.83	71.28	71.43
	Retired	57.67	57.43	67.63	67.43
	Student	81.92	81.01	83.90	82.91
	Others	65.57	65.27	63.89	63.63
Reference person professional status	Employee	68.49	68.47	73.58	73.58
	No employee	63.12	63.01	69.94	69.84
Gender	Man	71.23	71.20	74.36	74.43
	Woman	62.12	62.09	69.43	69.35
Age	16–34 years old	76.47	76.21	85.15	84.72
	35–49 years old	68.66	68.64	74.46	74.42
	50–64 years old	58.91	58.82	66.55	66.52
	65–74 years old	61.90	61.64	67.18	66.98
	75 or more years old	54.59	54.22	53.05	52.55
Nationality	Spanish	67.08	67.07	72.00	72.01
	Foreign	61.30	61.34	69.91	69.93
Marital status	Single and not living with a partner	68.70	68.62	71.74	71.98
	Married or single and living with a partner	66.20	66.21	72.45	72.43
	Widower, legally separated or divorced	64.73	64.52	68.83	68.58
Total		66.50	66.49	71.77	71.77

^(1)^Probabilities are expressed in percentages

**Table 6:** Odds-ratios, *Ω*_*i*/*j*_, from logit and probit models

		2006–07		2011–12	
Attribute	Alternative	Logit^(1)^	Probit^(2)^	Logit^(1)^	Probit^(2)^
Education level	No education or primary school (*i*)	-	-	-	-
	Secondary studies (*j*)	1.51	1.52	1.64	1.68
	University studies (*j*)	2.39	2.39	2.97	3.03
Interviewee’s professional status	Employee (*i*)	-	-	-	-
	Unemployed (*j*)	0.76	0.76	0.75	0.76
	Retired (*j*)	0.54	0.54	0.62	0.61
	Student (*j*)	2.06	1.97	1.73	1.65
	Others (*j*)	0.79	0.78	0.51	0.50
Reference person professional status	Employee (*i*)	-	-	-	-
	No employee (*j*)	0.76	0.75	0.81	0.80
Gender	Man (*i*)	-	-	-	-
	Woman (*j*)	0.61	0.61	0.74	0.73
Age	16–34 years old (*i*)	-	-	-	-
	35–49 years old (*j*)	0.65	0.65	0.49	0.49
	50–64 years old (*j*)	0.41	0.41	0.32	0.32
	65–74 years old (*j*)	0.47	0.46	0.33	0.33
	75 or more years old (*j*)	0.34	0.33	0.17	0.17
Nationality	Spanish (*i*)	-	-	-	-
	Foreign (*j*)	0.74	0.74	0.88	0.88
Marital status	Single and not living with a partner (*i*)	-	-	-	-
	Married or single and living with a partner (*j*)	0.87	0.88	1.04	1.03
	Widower, legally separated or divorced (*j*)	0.81	0.80	0.84	0.81

^(1)^In each attribute, the odds-ratios are expressed when the attribute category changes from *i* to *j*

^(2)^In the case of the probit model, the odds-ratios are calculated from average of individual odds-ratios when the attribute category changes from *i* to *j*

The two extreme categories of educational level produce differences in predicted average probabilities of reporting good health of 16–16.25 and 18.15–18.22 percentage points in each period, respectively. The odds-ratio between the probabilities of reporting good health and reporting not good health reveals that this effect has grown in relative terms, since in 2006–07 the odds-ratio for those with university studies is 2.1–2.4 times higher than for those with the lowest educational level, while in 2011–12 this ratio for the university studies is 2.5–3 times the one for the lowest educational level. On the other hand, the difference between employees and unemployed is about 5.2–5.4 and 4.6–4.9 percentage points in these two periods. If the reference person works, the interviewee’s likelihood of reporting good health exceeds the probability of those who live in households where reference person does not work by 5.4–5.5 and 3.6–3.7 percentage points in each period. Regarding demographic characteristics, men’s propensity to report good health exceeds women’s propensity by 9.11–9.12 and 4.93–5.08 percentage points in these two periods. The relative worsening of health among over 75 years old is also relevant. They do not follow the pattern of growing propensity to report good health that is observed in other age groups when comparing 2006–07 with 2011–12. In fact, the probabilities ratio for individuals over 75 is 2.7–3 times lower than for the youngest individuals in 2006–07 and more than 5–5.8 times lower in 2011–12.

**Fig. 1:**
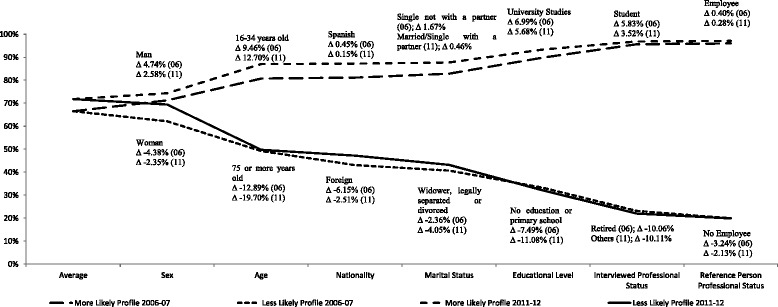
Extreme profiles’ average predicted probabilities of reporting good health from logit models ^(1)^

**Fig. 2:**
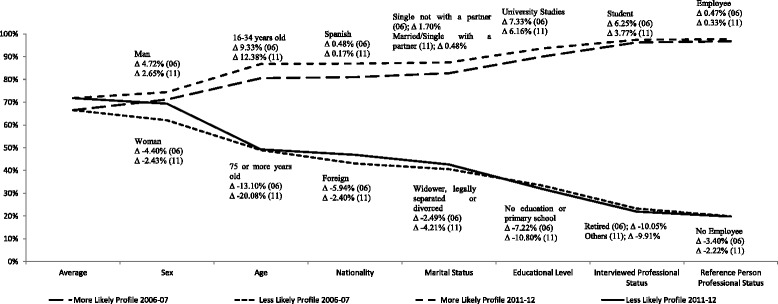
Extreme profiles’ average predicted probabilities of reporting good health from probit models ^(1)^

Finally, we have identified the individuals who present extreme profiles in terms of their propensity to report good health, with the aim to show the degree by which different socioeconomic and socio-demographic attributes contribute to generate health inequalities. Figures 1 and 2 reflect the marginal contribution of each attribute to the difference between extreme predicted probabilities as additional characteristics are incorporated until completing the identification of profiles with minimum and maximum propensity for 2006–07 and 2011–12. The first value of represented lines indicates the predicted average probabilities in each period. The next value represents the predicted average probabilities for each gender. Then, the predicted average probabilities corresponding to extreme profiles in terms of gender and age are evaluated, and so on until the rest of characteristics are incorporated. The final values of the respective lines indicate the difference in the predicted average probability of reporting good health for the extreme profiles, which represents 76.11–76.88 percentage points in 2006–07 and 77.29–77.98 percentage points in 2011–12.

## Discussion

Our results suggest that, despite the economic recession, an improvement in the Spaniards SAH has been observed (although it does not occur either between people with the lowest educational level nor between people aged 75 and over). However, while health inequalities by professional status and household economic situation have slightly reduced, those associated with educational level have increased. These are the main findings of the paper but we want to raise several points.

The samples of individuals considered in the research are clearly different with respect to their socioeconomic characteristics between 2006–07 and 2011–12 (see Table 1). For example, the percentage of people without studies or with primary studies has reduced from 41.92 % in 2006–07 to 22.56 % in 2011–12, whereas the percentage of people with secondary studies has increased from 41.10 to 61.49 % in the same period. Regarding the professional status of the interviewed, the reduction of the relative presence of employees (51.48 to. 45.00 %) is similar to the increase in the presence of no employees (7.17 to 14.60 %). Given that the economic situation of Spain worsened from one period to the other, it is foreseeable that changes in health inequalities are associated with changes in the socioeconomic status of residents. If this is the case, the improvement in SAH during a recession might be linked to these changing characteristics and would reflect patterns observed in other studies [16, 20, 26]. The improvement in individuals’ SAH with economic crisis periods has been reported elsewhere. According to López i Casasnovas, health can improve in recessive periods because the opportunity cost of having a healthy lifestyle is reduced and that associated with an unhealthy one is increased [32]. Ásgeirsdóttir et al. also observe that Icelanders’ lifestyles have improved during the current recession [33]. Astell and Feng, and Dávila and González argue that crisis periods can promote individual activities that contribute to capitalize health [19, 34]. However, other studies have found a worsening of health status associated with recessions [18, 19, 35, 36].

When we explore the different socioeconomic characteristics, different patterns are observed. With respect to professional status, employees are more likely than unemployed to report good health in both periods (see Table 5). Both groups experience an improvement in their SAH from 2006–07 to 2011–12, but the improvement is more pronounced for those unemployed, slightly reducing the difference in health between both groups during the recession. Similarly, Urbanos and González, using the same Spanish national health surveys (2006–07 and 2011–12) but restricting the sample to the working age population (16–65 years old), show that the percentage of Spanish unemployed who report bad health decreases more than among employees; in addition, they indicate that one or more years in a situation of unemployment have a negative impact on health (SAH and mental health) irrespective of the economic situation. Besides, the authors also conclude that SAH does not seem to worsen more with unemployment in times of economic crisis than before it [20]. Astell and Feng, in a study in Britain, detect worsening health that is more intensive among employees; among the reasons supporting this evidence, they point out the stress associated with fear of unemployment and job insecurity [19] and this may affect our measure of health (SAH). In addition, regarding those who are unemployed, it must be said that under poor economic expectations, investing in other human capital activities like education or health may become more attractive as time devoted to these activities has now a lower opportunity cost; this fact might help to explain partly the larger improvement in SAH of those unemployed during the recession period. Similarly, when we consider the household economic situation, we find that individuals who live in households where the reference person works –i.e. in a better economic situation- are more likely to report good health in both periods, but this difference in health is slightly attenuated in times of crisis (see Table 5). This result is in line with the reduction in health disparities by income level obtained by Kondo et al. [16].

The results regarding another socioeconomic characteristic as education level are rather different. The average predicted probabilities obtained in our study (Table 5) shows that the propensity to report good health increases from 2006–07 to 2011–12 for any educational level considered. However, such propensity is wider for those individuals with university studies than for those with primary studies; as a consequence, differences in health by educational level are increased during the crisis. The positive effect of education on health is well reported in the literature (for a review of related theories and evidence see Cutler and Lleras-Muney [37]). Better educated individuals are less likely to report that they are in poor health, probably in part due to their behaviour and skills as compared with individuals with primary or no education level. Better educated individuals have relatively healthier behaviour regarding smoking, drinking, exercise, etc. When health care is needed, it is also assumed that they can manage more efficiently the use of health care and preventive services (in this case, within the Spanish National Health System services, which is based on the equality of access principle). All these activities are driven by resources. In a context of economic difficulties and austerity policies, there is less availability of these resources, so in our opinion, the skills and more information of those more educated individuals may help them to adapt better to economic hardship and as a result to get health improvements more effectively in the margin.

Regarding other factors like gender and age that we have controlled for in this research, we must say that there is an increase in the likelihood of reporting good health by women and men from 2006–07 to 2011–12, which is more pronounced among women (see Table 5). Thus, the difference in health by gender is reduced during recession, the opposite to the findings of Kondo et al. [16]. Borrell et al. consider that gender produces health inequalities [2], since men and women have different characteristics (biological, physical, cultural, socioeconomic,…). In addition, women are one of the more vulnerable groups to recessions, since they start from worst socioeconomic conditions before crises, which are then exacerbated during them [38] (lower qualification/authority grade jobs [28], greater difficulties to access job market, lower wages,…).

In addition, attributing differences in health to groups with different socioeconomic profiles can be influenced by an age effect, which the statistical model is not always able to isolate. Individuals with low educational levels and retirees are mainly older people, while the relative weight of younger people is higher among those with university studies or students. According to predicted probabilities shown in Table 5, the propensity to report good health descends from 2006–07 to 2011–12 among older individuals –i.e. among individuals who need more health care-. However, although the age effect is taken into account when logit or probit models are estimated, according to these estimates a slightly reduction in such a propensity is also observed among individuals without studies or with primary studies. Predicted probabilities of the extreme socioeconomic profiles allow us to assess to what extent the explanatory factors joint action can exacerbate or mitigate health inequalities when an economic crisis takes place. Although living conditions of more disadvantaged people worsened during a crisis, their propensity to report good health has been maintained, while the propensity to report good health of the most advantaged profile has increased. Therefore, differences in health between both profiles have slightly widened during the recession.

Our study is not exempt from some limitations. First, results are conditioned because of the way in which SAH response alternatives are grouped, which could imply loss of information. Second, endogeneity problems could exist and estimations could show an apparent effect of socioeconomic status on SAH that is, in part, a reflection of the impact of health on the socioeconomic status. Third, the proportion of interviewees who are the household reference person is high, so both of the attributes, interviewee’s professional status and household’s economic situation, could contain similar information. And, fourth, the statistical model used is not always able to isolate the effect of some attributes on certain groups’ propensity to report good health.

## Conclusions

With the caution corresponding to the limitations mentioned, we conclude that the effect of socioeconomic status on SAH behaves differently during a crisis and, also, depends on the socioeconomic status indicator considered. In times of crisis, differences in SAH by educational level are amplified while those linked to professional status and household economic situation are reduced. Additionally, once controlled by age, educational level is the socioeconomic attribute that produces the greatest differences in health both in periods of growth and recession. The design of public policies aimed to prevent a worsening of health inequalities during recession periods should take into account these differential effects of socioeconomic status indicators on health inequalities.

## Appendix

The discrete changes *DC*(*q*, *r*) are a way of measuring the average change in the probability to report a good health status when the pair of explanatory variables (*x*_*m*_, *x*_*n*_) moves from (*x*_*m*_, *x*_*n*_)^*q*^ to (*x*_*m*_, *x*_*n*_)^*r*^, that is to say,

$$ DC\left(q,r\right)={\displaystyle \sum_i\frac{w_i}{{\displaystyle \sum_i{w}_i}}P\left(SA{H}_i=1/{x_i}^q\right)}-{\displaystyle \sum_i\frac{w_i}{{\displaystyle \sum_i{w}_i}}P\left(SA{H}_i=1/{x_i}^r\right)}, $$

where *w*_*i*_ is the weight factor assigned to individual *i* and *x*_*i*_^*q*^ and *x*_*i*_^*r*^ are the vectors of explanatory variables for individual *i* when it is assumed that (*x*_*m*_, *x*_*n*_) = (*x*_*m*_, *x*_*n*_)^*q*^ or (*x*_*m*_, *x*_*n*_) = (*x*_*m*_, *x*_*n*_)^*r*^, respectively. Note that a change in the category of one of the attributes in Table 1 (gender, age, nationality or marital status) implies a change in two dichotomous variables. In spite of that, discrete changes can also be calculated when comparing individuals whose profiles differ in various characteristics.

On the other hand, the odds-ratios are defined as

$$ \varOmega =\frac{P\left(SA{H}_i=1/{x}_i\right)}{P\left(SA{H}_i=0/{x}_i\right)}, $$

whereas the quotient of these ratios when the vector of explanatory variables *x* moves from *x*^*q*^ to *x*^*r*^ can be expressed for the logit model as.

$$ \varOmega \left(q,r\right)=\frac{\varOmega (q)}{\varOmega (r)}={e}^{\beta^{\mathit{\hbox{'}}}\left({x}^q-{x}^r\right)}. $$

Note that, in this case, the quotient of the odds-ratios when one of the individual characteristics changes category does not depend on the remainder of individual characteristics. Therefore, the quotient of the odds-ratios when several individual characteristics are modified can be directly deducted as a product of the corresponding quotients of the odds-ratios when only one of these characteristics is changed. On the other hand, for the probit model, odds-ratios are not constant, but an useful approach for each attribute can be calculated as a weighted average of individual odds-ratios when the attribute category changes from *i* to *j*.

## Abbreviations

SESPAS: Spanish Society for Public Health and Health Administration
NHS: National Health Survey
INE: National Statistics Institute
MSSSI: Spanish Ministry of Health, Social Services and Equality
SAH: Self-Assessed Health
SPSS: Statistical Package for the Social Sciences
